# Cochlear Implant Research and Development in the Twenty-first Century: A Critical Update

**DOI:** 10.1007/s10162-021-00811-5

**Published:** 2021-08-25

**Authors:** Robert P. Carlyon, Tobias Goehring

**Affiliations:** Cambridge Hearing Group, MRC Cognition & Brain Sciences Unit, University of Cambridge, Cambridge, CB2 7EF UK

**Keywords:** Cochlear implants, Plasticity, Pitch, Hearing

## Abstract

Cochlear implants (CIs) are the world’s most successful sensory prosthesis and have been the subject of intense research and development in recent decades. We critically review the progress in CI research, and its success in improving patient outcomes, from the turn of the century to the present day. The review focuses on the processing, stimulation, and audiological methods that have been used to try to improve speech perception by human CI listeners, and on fundamental new insights in the response of the auditory system to electrical stimulation. The introduction of directional microphones and of new noise reduction and pre-processing algorithms has produced robust and sometimes substantial improvements. Novel speech-processing algorithms, the use of current-focusing methods, and individualised (patient-by-patient) deactivation of subsets of electrodes have produced more modest improvements. We argue that incremental advances have and will continue to be made, that collectively these may substantially improve patient outcomes, but that the modest size of each individual advance will require greater attention to experimental design and power. We also briefly discuss the potential and limitations of promising technologies that are currently being developed in animal models, and suggest strategies for researchers to collectively maximise the potential of CIs to improve hearing in a wide range of listening situations.

## INTRODUCTION

### Overview

Cochlear implants (CIs) are the world’s most successful sensory prostheses, having restored hearing to more than 800,000 deaf people worldwide and providing improved speech perception to the majority of them (Boisvert et al. 2020). They also provide a remarkable scientific opportunity; for example, they allow one to control stimuli in a way that is unaffected by cochlear processing and to study the changes that occur in the auditory system when sensation is restored after a long period of deprivation. Their clinical success and scientific potential have been accompanied by an explosion of research activity in the field, with more than 15,000 articles since the turn of the century including the word “cochlear implant” in the title or topic list. However, it can be argued that the major clinical and scientific advances were achieved before this arbitrary time point. Here we evaluate both the clinical and scientific progress that has been achieved by CI research since the year 2000, consider reasons for the limitations in the success of this endeavour, and suggest ways in which researchers can in future take full advantage of the advances that have been made.

**Fig. 1:**
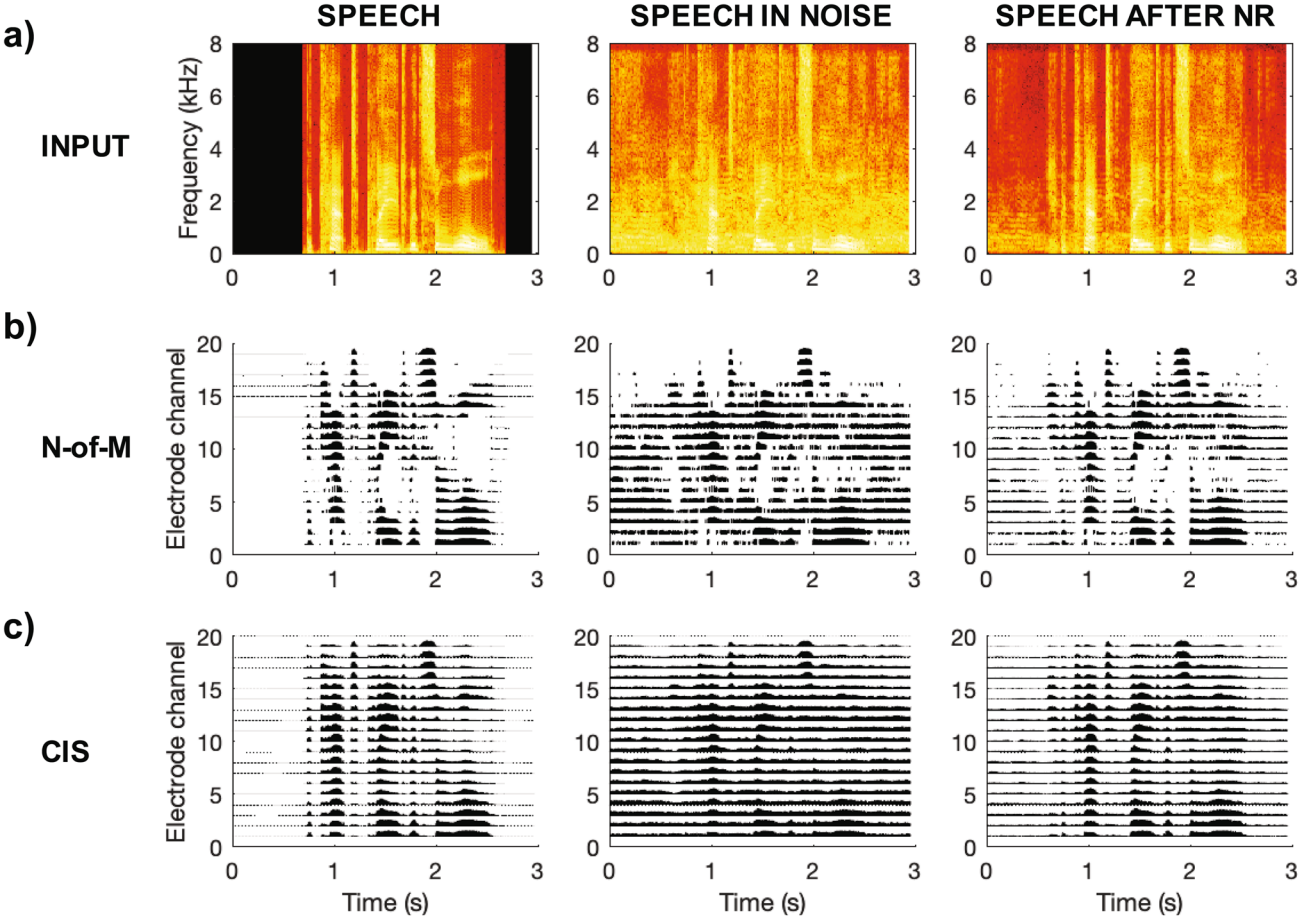
Part **a**) shows the spectrograms of the sentence “The cat played with some wool.” in quiet, in a 20-talker babble noise at a signal-to-noise ratio of 10 dB, and after processing with a DNN noise reduction (NR) algorithm (Goehring et al. 2019a). Parts **b**) and **c**) show the electrical stimulation patterns (electrodograms) after processing with a 20-channel n-of-m strategy with 8 maxima and with a 20-channel CIS strategy, respectively

This review starts with the external components of the CI and the pre-processing that they perform, before working inwards towards the brain. At the turn of the century, behind-the-ear processors and microphones had only recently been introduced and most CI listeners still used body-worn devices. Directional microphone algorithms were not used and external processors did not incorporate sophisticated signal processing such as noise reduction. Our first section therefore considers the advances made in microphones, noise reduction, and further pre-processing strategies. Our second section considers the development of new coding algorithms used to transform acoustic signals to electrical stimulation patterns, which, in the year 2000, consisted primarily of the Continuous Interleaved Sampling (CIS) and n-of-m strategies (Fig. 1). In doing so, we evaluate published comparisons between strategies that have been implemented in clinical use, as well as comparisons between newly proposed experimental strategies and the standard clinical ones. The third section then considers the effects of new stimulation configurations designed to produce more focused current spread within the cochlea. The limited success of these “one size fits all” changes to speech-processing strategies and stimulation modes has led to increased interest in an alternative approach, which is to make adjustments on a patient-by-patient basis. One basic insight is that the so-called electrode-neural interface (ENI), comprising factors such as the position of the electrode and the functional status of the auditory nerve, can differ substantially not only across listeners but also between CI electrodes in the same listener. The success of these bespoke methods, which include turning off or reprogramming subsets of electrodes, is the subject of the fourth section. Our fifth section considers the advances made in our understanding of auditory processing; this includes insights gained into basic sensory processes and into the plastic changes and auditory learning that occur after auditory sensation has been restored by a CI. Our final section starts with an overview of the advances that have been made in CI research and development this century. We then summarise the dramatic increase in the range of patients and in applications of CIs, since the turn of the century; these include the implantation of babies and children, implantation of people with residual acoustic hearing, and the increased prevalence of binaural implantation. We then discuss the extent to which these advances could have been achieved with the knowledge and technology available at the turn of the century. We conclude with a discussion of the success of research and development in the last two decades and with some recommendations for the future.

### Scope of the Review and Selection of Evidence Reviewed

Broadly speaking, this review concerns the evaluation of methods that have been used in an attempt to improve hearing by human CI listeners, followed by a description of fundamental scientific advances in the understanding of the response of the auditory system to electrical stimulation. In addition, the “Discussion” section briefly considers promising new technologies, such as optogenetic stimulation, intra-neural stimulating electrodes, and methods to minimise or reverse neural loss, that have so far been evaluated in animals. Because the application of those methods to human CI listeners lies in the future, we primarily refer the reader to comprehensive and recent reviews by specialists in those fields. We do not discuss developments in surgical techniques, which lie beyond our area of expertise, and that have been recently reviewed elsewhere (Nguyen et al. 2016; Dhanasingh and Jolly 2017; Khater and El-Anwar 2017; Bruce and Todt 2018).

The reader will be relieved to learn that, even within these constraints, we do not review every CI article published this century. The remainder of this subsection describes some of the general criteria we have used for inclusion, and briefly discusses the desired and actual characteristics of CI studies.

In a perfect world, an experimental evaluation of a novel CI development would have a number of desirable features. To aid interpretation of the results, only one thing would be varied at a time. To avoid false positives, testing would be performed double blind and there would be a plausible control group or treatment. Sample sizes would be calculated so as to guarantee adequate statistical power according to the effects of interest. Further statistical treatment of the results would control for multiple comparisons not only in terms of the independent variable (e.g. the number of different signal processing strategies being compared) but also in terms of the dependent variables, such as the number of different speech tests being performed. Where performance is evaluated using just one test, for example with a particular type of masker, the authors would consider whether a similar benefit would be observed with other masker types and test materials. In longitudinal studies, care would be taken when interpreting improvements over time, so as to rule out learning effects that may arise from the participants becoming acquainted with the test materials. To avoid false negatives, participants would be given sufficient time to acclimatise to a novel intervention.

Unfortunately, the world is not perfect. (This section is being written in 2020/2021.) Some deviations from the ideal scenario are inevitable, or at least hard to overcome. New versions of CIs may incorporate multiple changes, for example in the processing strategy, automatic gain control, and the range of frequencies analysed. Participants may recognise their everyday speech-processing strategy and notice when they have been given a new one, even if the experimenter does not tell them. The experimenter may not have a clear hypothesis about which situation(s) will reveal the benefits of a particular intervention, and so use multiple tests; simple (e.g. Bonferroni) but conservative correction for multiple comparisons would reduce the power of studies that obtain a wide range of outcome measures. Statistical power is frequently constrained by the limited number of CI participants available to the researcher. Giving participants as much experience with a novel device or algorithm as they have with their existing clinical device is time-consuming, practically infeasible, and could, if the intervention is not successful, expose participants to several months of poor hearing, or even result in maladaptive plasticity.

As a result of these necessary limitations, it is hard to find a study that unambiguously identifies an improvement that applies to a wide range of target speech and masker types. Nevertheless, it is clear that some studies have made more efforts to overcome these problems than others. When deciding which studies to discuss in detail, we have tended to include those that, in our opinion, have most successfully overcome the pitfalls described above; this necessarily involves pointing out the inevitable limitations. A second criterion has been to include studies that have proved exceptionally influential. We have also tried to include the most recent research, especially where the most recent reviews of the topic are several years old. We focus our review of evaluation studies on those that measure speech perception, because of its overwhelming importance for education, employment, and social interactions. In the fifth section, where we consider the basic scientific advances obtained from CI research, we have attempted to identify “reliable surprises”. We define these as findings that were not a priori predictable and that have been replicated in two or more published articles, preferably from different research groups.

## PRE-PROCESSING STRATEGIES

CIs pick up speech and environmental sounds via one or more integrated microphones located in the speech processor. The recorded acoustic signals are then sampled and converted to the digital domain for pre-processing. Several pre-processing methods are commonly used to improve the signal-to-noise (SNR) ratio between “direct” speech from a target talker and background noise and/or reverberation. These methods include multi-microphone processing for spatial filtering (often called “directional microphones” or “beamformers”), single-channel noise reduction, and further strategies specifically for dereverberation and speech enhancement. Most of these strategies have been inspired by methods used or developed for acoustic hearing aids and that benefit from a longer tradition of research and development than for CIs. Since the turn of the century, several hearing aid manufacturers have teamed up with cochlear implant manufacturers to benefit from synergies in the overlapping goals for pre-processing strategies. In principle, any improvement in signal-to-noise ratio (SNR) with pre-processing strategies can be expected to be valuable for CI listeners, due to their difficulties when listening to speech in noisy and reverberant settings. Furthermore, benefits with pre-processing strategies may be more attainable with CIs than with hearing aids, because speech-processing algorithms generally function better at the high SNRs often necessary for speech perception by CI users in real-world situations (Wu et al. 2018). However, these assumptions are not proven or generalisable and differences in speech perception with CIs compared to acoustic hearing warrant further evaluation via CI listening studies.

### Directional Microphones

Directional microphones (DMs) receive signals from multiple (often two) omnidirectional microphones and combine them into a single-channel signal by making use of spatial differences between microphones. The omnidirectional microphone signals are delayed, weighted, and summed to generate directional (e.g. hypercardioid) patterns and to enhance the SNR of signals from certain directions. Adaptive filters are often used to improve the directional characteristics further. In order to improve speech perception, DM strategies therefore rely on the assumption that speech and noise signals are spatially separated. For conditions that fulfil this assumption (e.g. target speech in front and noise at one side of the listener), several studies have reported significant improvements in terms of correctly identified sentences (van Hoesel and Clark 1995; Chung et al. 2006; Chung and Zeng 2009) and speech reception threshold (SRT), defined as the SNR required for 50% correct performance (Wouters and Vanden Berghe 2001; Spriet et al. 2007; Hersbach et al. 2012; Baumgartel et al. 2015). The first application of adaptive beamformers in CIs led to improvements of between 3.7 and 16 dB in SRT, which can be considered very substantial, and have been obtained using single-blinded (Chung et al. 2006; Chung and Zeng 2009) or double-blinded designs (Spriet et al. 2007). However, in conditions with multiple interfering noise sources or reverberation, the sound field becomes more diffuse and the benefit of DM strategies is reduced compared to conditions with clear spatial separation between sources and no reverberation (Baumgartel et al. 2015). Hersbach et al. (2013) developed an improved version of an adaptive directional microphone that achieved significant benefits in more diffuse environments with moving noise sources (that were still spatially separated from the speech signal). That study did not mention whether or not blinding was used; for conciseness throughout this review, we make the uncharitable but realistic assumption that if blinding was not mentioned then it was not performed. As mentioned above, directional algorithms can only provide benefits when the speech and noise signals are not co-located but they have the advantage of performing well in any kind of spatially separated noise.

### Noise Reduction

Single-channel noise reduction (NR) strategies operate as if a single microphone were used for the recording, and do not rely on spatial information or differences. Current CI devices use NR techniques based on traditional signal processing approaches and make specific assumptions about statistical differences between speech and noise signals. These approaches first estimate the power spectrum of the background noise (e.g. by using the rear-facing, rejected signal from directional filtering; Hersbach et al. 2013) and then estimate an *a priori* SNR in each time–frequency unit. These SNRs are then used to weight the spectro-temporal representation of the noisy speech with a gain function that retains speech-dominated but not noise-dominated parts of the signal. NR processing leads to an *enhanced* speech signal that ideally contains less background noise than before the filtering. Several studies investigated these traditional NR approaches and found significant improvements in speech perception for CI listeners (Loizou et al. 2005; Hu et al. 2007; Dawson et al. 2011; Mauger et al. 2012; Ye et al. 2013; Chen et al. 2015; Wang and Hansen 2018). Improvements ranged up to 2 dB in SRT or 25% in percentage correct, much smaller than those reported for directional microphones, and were mostly observed for stationary background noise. In modulated, non-stationary noise, such as multi-talker babble, traditional NR approaches are often even less successful or completely fail to improve speech intelligibility due to the absence of differences between speech and background noise statistics.

More recently, a new class of NR algorithms based on machine-learning (ML) techniques, such as deep neural networks (DNNs) or Gaussian mixture models, have proved more successful in enhancing the intelligibility of speech in noise for CI listeners (Hu and Loizou 2010; Goehring et al. 2017, 2019a; Lai et al. 2018). Some of these studies used double-blinded designs, self-administered testing without experimenter involvement, and/or adaptive-SRT testing that would make it more difficult for the listener to identify the condition under test than with fixed SNR testing (Goehring et al. 2017, 2019a). Improvements (reductions) in SRT were somewhat larger than with traditional techniques and ranged from 1.4 to 6.4 dB depending on the background noise. Importantly, there was some success for non-stationary noise when the algorithm was optimised for a specific type of noise. For example, Goehring et al. (2019a) reported improvements when the background was multi-talker babble but not when it was factory noise (example stimuli shown in Fig. 1). The main concept is based on the ideal ratio or binary mask, which requires prior information about the signal and noise when presented separately, and adjusts the energy in each time–frequency segment of the mixture so as to maximise the overall SNR. ML models are then trained on acoustic data to estimate these ideal masks and subsequently process the speech in noise using the estimated masks. However, as ML-based NR strategies rely on limited training data, there remains the challenge of generalisation to acoustic conditions (e.g. different voices and background noises) that deviate from the ones used during model training. A large mismatch between training and testing conditions reduces DNN performance and intelligibility benefits (Goehring et al. 2017), most likely due to estimation errors that compromise the removal of noise in speech gaps and the preservation of speech transients (Kressner et al. 2019). Such insights into the error patterns that are most limiting to performance motivate future research to optimise these techniques further and incorporate the requirements of CIs and of individual listeners. For example, CI listeners may benefit from and prefer more aggressive noise reduction settings in some listening conditions than the standard settings used in noise reduction systems (Mauger et al. 2012), and the introduction of speech distortions due to noise reduction may not be greatly detrimental as long as channel selection remains intact (Qazi et al. 2013).

Using DM and NR strategies in sequence has been shown to provide significantly better outcomes than with the individual strategies alone (Buechner et al. 2011; Hersbach et al. 2012, 2013; Baumgartel et al. 2015). It should be noted that these results were obtained for DM strategies combined with traditional NR strategies and there is potential to obtain even larger benefits by using ML-based NR techniques in combination with DM strategies and by using optimised models for specific acoustic environments. Continued efforts in ML and speech research have delivered steady success in making ML-based NR approaches more robust, memory-efficient, and capable of working in real time, but it remains to be seen when this technology will find its way into actual CI devices.

### Other Pre-processing Strategies

Further pre-processing strategies for CIs have been developed to improve speech perception in reverberant environments. These were first based on ideal strategies (Kokkinakis et al. 2011) that use the speech-to-reverberation ratio (SRR) instead of the SNR to generate an ideal reverberant mask for speech dereverberation based on ground-truth information about speech and reverberation (as with the ideal binary mask for noise removal). This method attenuates those parts of the signal that are dominated by reverberation and retains the parts that are dominated by speech. Again, due to the ideal nature of this approach, improvements of up to 65% in speech intelligibility have been observed. Hazrati et al. (2013) then tested a “blind” binary reverberation mask, that was based on variance-based features (e.g. kurtosis) together with an adaptive thresholding method without access to the clean speech signal, and reported smaller but still significant improvements of 23% and 27% in relatively high reverberation conditions (reverberation times of 0.6 and 0.8 s, respectively).

### Summary

Overall, there is potential for large benefits to speech perception in noisy situations with DM approaches, but only if the speech and noise are somewhat spatially separated. Traditional single-channel noise reduction approaches do not require spatial separation but have yielded smaller and very limited improvements; however, ML-based methods have provided a strong performance boost to this type of processing. Ultimately, the combination of smart DM techniques with ML-powered noise reduction and dereverberation methods constitutes the most promising prospect of further improvement to CI pre-processing strategies, and could be delivered to new and existing CI listeners via an upgrade of their external CI speech processor. An advantage for the development of CI pre-processing strategies is that objective measures (such as SNR improvement and algorithms for speech intelligibility prediction) can be used to assess the efficacy of novel techniques in comparison to previous methods together with CI listening tests. While it may not be possible to fully blind the listener to DM or NR processing, due to the changes of the SNR or the introduction of processing artefacts, rigorous attempts should be made to avoid biases, especially on the experimenter side. Another aspect concerns the possibility that CI listeners may acclimatise to pre-processing strategies, as they do with CI coding strategies. Most of the studies reviewed in this section used acute testing conditions with interleaved, randomised presentation of stimuli that would not allow for such adaptation. This means there is potential for further benefits if CI listeners are allowed to adapt and compensate for the typical speech distortions and noise artefacts introduced by DM, NR, and dereverberation pre-processing approaches. Finally, any benefits provided by pre-processing strategies are expected to occur regardless of later CI processing stages, and therefore provide a means of improving speech outcomes for all listeners irrespective of their device, processing strategy, or ENI.

## SIGNAL PROCESSING STRATEGIES AND NEW STIMULATION METHODS

### Commercially Available Strategies

As noted in the Introduction, turn-of-the-century signal processing strategies consisted primarily of the CIS or n-of-m strategies (Fig. 1). Both of these strategies pass the signal through a bank of bandpass filters, extract the envelope at the output of each filter, and use these envelopes to amplitude modulate fixed-rate pulse trains presented on each electrode. Pulses on different electrodes are interleaved in time so as to minimise between-electrode charge interactions. Although a strategy that used analogue rather than pulse-train carriers was still available in the Advanced Bionics (AB) implant, most users of the AB and MedEl implants used a standard CIS strategy, whereas an n-of-m strategy, termed SPEAK, was widely implemented in the Cochlear device. A new strategy, termed ACE, was introduced around the year 2000, and differed from SPEAK primarily by increasing the pulse rate from 250 to 900 pulses-per-second (pps) in each channel.

The introduction of ACE represents the most widely implemented change in strategies used by the Cochlear device this century, and so it is important to know whether it provides advantages compared both to SPEAK and to CIS. Skinner et al. (2002) compared speech perception scores between SPEAK, ACE, and CIS in a group of twelve newly implanted listeners, thereby avoiding complications due to participants being experienced with one of the strategies under test. A further strength was that the participants (although not the experimenters) were blinded to the condition being tested. Scores for sentences in eight-talker babble were slightly and significantly higher for ACE than for SPEAK (8.8%) and CIS (5.6%). However, although there were also some benefits for the perception of isolated words, identification of vowels in a consonant–vowel-consonant (/cVc/) context was significantly *worse* by 7–8% for ACE than for SPEAK. Kiefer et al. (2001) also compared SPEAK, ACE, and CIS, this time for more experienced CI users, and reported benefits for ACE compared to both CIS and SPEAK. However, in that study, the order of testing was not fully counterbalanced between the strategies, such that ACE was never tested first, and so one cannot rule out the influence of practice effects (cf. de Jong et al. 2019). Psarros et al. (2002) reported significant improvements in word identification in quiet when seven children were converted from the SPEAK to the ACE strategy, and, importantly, found that performance deteriorated again when they were switched back to SPEAK. However, testing was not blinded, and, as the authors pointed out, any influence of strategy on masked sentence identification was obscured by strong learning effects, as evidenced by better performance on the second compared to the first test of the SPEAK strategy. Overall, a reasonable conclusion is that there is little evidence for substantial overall differences between the ACE, CIS, and SPEAK strategies, but that thereis some evidence that performance on the ACE strategy may be slightly superior for some tests. A more recent commercially implemented modification to ACE is the MP3000 strategy, which, in each brief (20 ms) time frame, identifies and removes pulses that would be masked by higher-amplitude pulses presented on other channels. This has been shown to reduce power consumption but did not improve speech perception in a clinical trial (Buechner et al. 2011).

The major qualitative change in CIS strategies has been the introduction of coding of the temporal fine structure into the pattern of electrical stimulation. These and other changes to speech-processing strategies have been comprehensively reviewed by Wouters et al. (2015). MedEl developed the fine structure processing (FSP) strategy, which, in the lowest-frequency channels, codes each zero crossing of the filtered waveform with short bursts of pulses, with the remaining channels using a high-pulse-rate CIS strategy. Subsequent modifications to the FSP strategy differed primarily in the number of low-frequency channels that coded fine-structure information; for example, the FS4 strategy applies FSP to the lowest four channels compared to two in the previous version. Advanced Bionics devices also code temporal fine structure in their HiRes, HiRes120, and subsequent strategies.

A large number of studies have investigated the benefits of fine structure processing, as implemented in the MedEl device (Hochmair et al. 2015). One complicating factor is that changes from CIS-based to FSP-strategies have, in many of these studies, been accompanied by increases in the frequency range of the analysis bands. One double-blind study that compared FS4 to HDCIS, which is a high-pulse-rate version of CIS, while using the same (extended) frequency range for the two found no difference between them (Riss et al. 2016). Two widely cited articles that reported benefits for FSP compared to CIS, and that used the same frequency range for each strategy, were published by Vermeire et al. (2010) and by Kleine Punt et al. (2014). Both articles followed the same group of 22 participants who were switched from the Tempo + processor, which used a CIS-based strategy, to the Opus speech processor which was programmed with an FSP strategy and with an extended frequency range (CIS +). A further 10 participants were switched to the Opus processor but continued to use a CIS-based strategy, albeit with an extended frequency range, because the fitting software deemed them “unable to benefit from FSP processing”. Vermeire et al. measured SRTs for sentences in noise at baseline (prior to the change), at switchover, and at 1, 3, 6, and 12 months. They noted that only the FSP group improved significantly from baseline to the 12-month measure (although they did not perform the more appropriate test of whether the improvement was significantly greater than for the CIS + group). However, importantly, the FSP group also improved significantly at 12 months when re-tested using the CIS + strategy, consistent with the improvement arising from a learning effect, such as increased familiarisation with test materials. Kleine Punt et al. (2014) reported SRTs obtained additionally at 24 months and found that the SRT had dropped by a further 6 dB for the FSP group. They did not test whether, as occurred at the 12-month time point, this substantial improvement would also be observed if participants were re-tested on the CIS + strategy; this would have been helpful so as to rule out learning effects.

Buechner et al. (2006) performed a retrospective comparison of patients switched from previous strategies (including CIS) to the HiRes strategy and reported some improvements, but the interpretation of the results is complicated by possible learning effects and the fact that the HiRes strategy used 16 channels compared to 8 channels in the other strategies. Overall, evaluations of the strategies that incorporate temporal fine structure have produced mixed results, with no strong evidence for a consistent benefit (Magnusson, 2011b; Riss et al. 2011, 2016; Muller et al. 2012). As Wouters et al. (2015) have pointed out, one reason for this may be because the fine structure is not always in phase in all frequency channels, so that, for example, two tones separated by an octave will produce non-aligned pulse trains in different channels. Even though these two tones may primarily excite different electrodes, current spread within the cochlea means that each neuron will respond to mixtures of these misaligned pulses, leading to a complex or unclear pitch. Another likely reason is the biological limitation on temporal processing by CI listeners, manifested in the finding that, even with simple pulse trains applied to a single electrode, discrimination of rate changes is poorer than for normal hearing and deteriorates dramatically at rates higher than about 300 pps (Townshend et al. 1987; Shannon and Otto 1990; Carlyon et al. 2008; Kong et al. 2009).

### Experimental Strategies

One approach to improving commercial speech-processing algorithms has been to add a stage that enhances the modulations present in each channel. Two such methods, eTone and F0Mod, identify voiced portions of speech and apply amplitude modulation at the estimated fundamental frequency (F0) with subsequent processing identical to ACE. Recent real-time implementations of these strategies did not reveal any improvements in speech perception relative to ACE, although there was some evidence that F0Mod could improve F0 discrimination of harmonic complexes (Francart et al. 2015; Vandali et al. 2019). This strategy was also shown to improve pitch and melody judgements in two unblinded studies (Laneau et al. 2006; Milczynski et al. 2009). Another envelope enhancement strategy subtracts a 20-Hz low-pass filtered version of the envelope in each channel from the un-filtered envelope, thereby enhancing onsets in the speech envelope. Koning and Wouters (2016) recently reported a small (1 dB) but significant improvement in the SRT for speech masked by a single talker and for four participants, compared to that for ACE, using a double-blind design. More recently, Lamping et al. (2020) developed a strategy that passes the envelope in each channel through a temporal window that has been used to model masking in both normal-hearing and CI listeners, and, in each channel, deletes pulses that are likely to be masked. This “Temporal Integrator Processing Strategy (TIPS)” enhances the envelope modulations in each channel and may reduce unwanted charge interactions between pulses in nearby channels. Lamping et al. (2020) reported that adding the TIPS processing stage to the CIS strategy produced an average of 2.4 dB improvement in the SRT for sentences masked by stationary noise for eight participants, and suggested that it could reduce power consumption substantially. Experimenters but not participants were aware of which condition was being tested. The speech test used (Wagener et al. 2003) was scored automatically, thereby minimising but not necessarily eliminating experimenter effects. Kludt et al. (2021) also developed a strategy based on a temporal masking model and reported improvements of 10–11% in the intelligibility of speech in stationary noise over the MP3000 strategy, but the authors did not report whether participants and/or experimenters were blinded as to which conditions were being tested.

Another method is to enhance the representation of the signal spectrum by modifying the pattern of stimulation across electrodes at each time point. Two such strategies do so by adding a processing stage that reduces the tendency of ACE to select “clumps” of adjacent electrodes. Both have produced small, significant improvements using double-blind counterbalanced designs. Nogueira et al. (2016) introduced the Spectral Enhancement Strategy (SES), which attenuates energy in the spectral valleys prior to channel selection, and reported a 0.57 dB improvement in SRT relative to ACE. Bolner et al. (2020) described another method, termed SPACE, that compensates for the estimated current spread from each electrode prior to the channel selection stage. When applied to mixtures of target speech in four-talker babble, without prior knowledge of the clean speech, it produced a significant 1.4 dB reduction in SRT averaged across six participants, compared to the standard ACE strategy. However, no significant advantage was observed for speech in stationary noise.

**Fig. 2:**
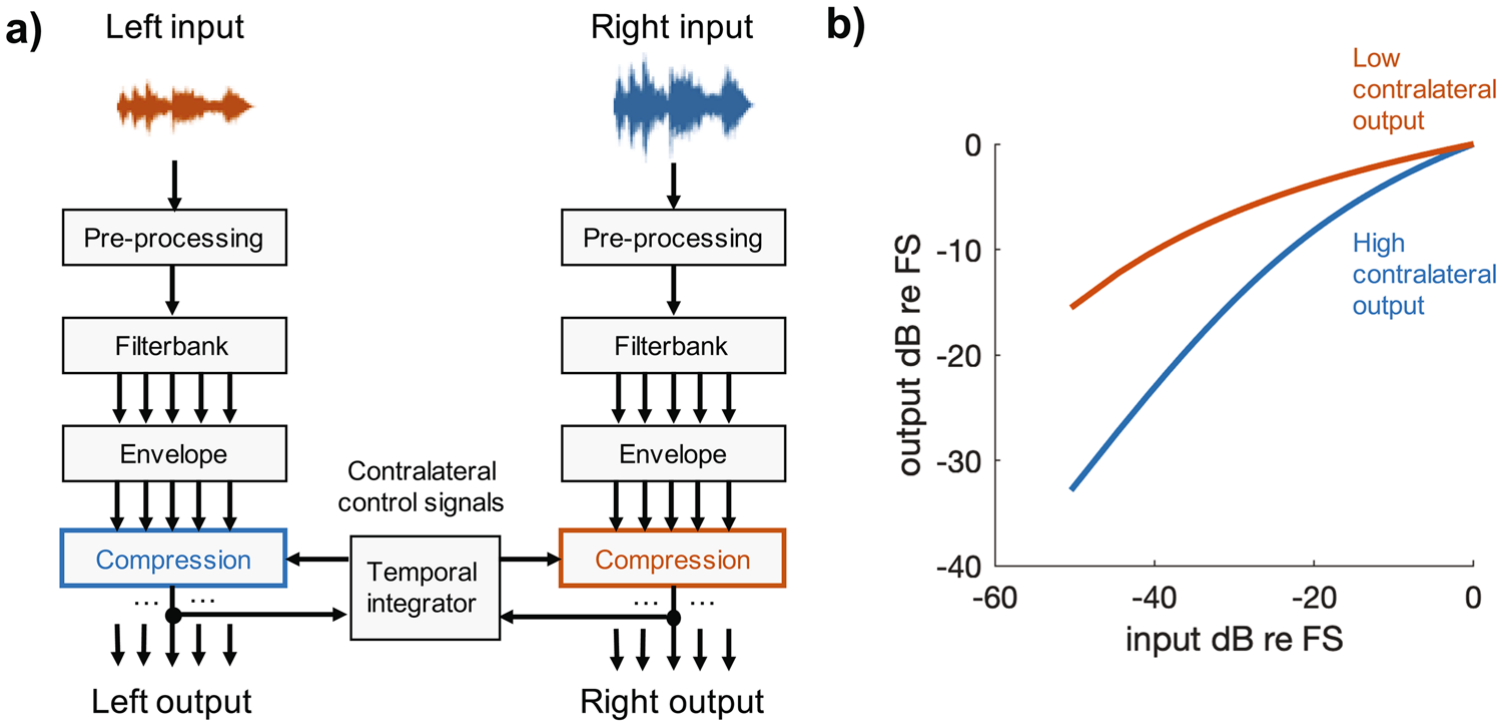
**a**) Schematic of the processing diagram used by the MOC strategy described in the text and introduced by Lopez-Poveda et al. (2016). **b**) Example of two input–output functions for the same channel under conditions where the recent output of the corresponding contralateral channel is high or low. A high contralateral output causes the input–output function to become more compressive

The experimental strategies described above, as well as those in everyday clinical use, were designed to work for unilaterally implanted patients; they can of course be used by bilateral recipients but operate independently at each ear. More recently, Lopez-Poveda and colleagues introduced a strategy that incorporates communication and processing between the two speech processors of bilateral CI recipients (Lopez-Poveda et al. 2016, 2017, 2019, 2020; Lopez-Poveda and Eustaquio-Martin 2018). This “MOC” strategy was inspired by the medial olivocochlear reflex that operates in acoustic hearing but not with CI stimulation, and adds additional processing at the front end of the standard CIS algorithm. Whereas in CIS the envelope in each channel is passed through a fixed compressive nonlinearity, the input–output function in any one channel of the MOC strategy depends on the output level of the corresponding channel in the other ear. This is done in such a way that, when the output of a frequency channel in the processor on one ear has a high amplitude, the input–output function in the corresponding channel of the opposite ear changes so as to become more linear and to reduce the gain applied to low-level inputs (Fig. 2). In this way, when the stimuli reaching the two ears are different, spectral peaks at one ear may attenuate the representation of spectral dips in the other, and vice versa*.* The latest version of the algorithm (MOC3) has been shown to produce a 1–2 dB improvement in SRT *re* CIS for speech masked by stationary noise, both when the speech and noise are spatially separated and when they are both presented straight ahead of the listener (Lopez-Poveda et al. 2020). In the latter situation, the power spectra at the two processors would be identical, and so the improvement of SRT presumably arises from an identical attenuation of low-level portions of the signal at the two ears. An interesting check would be to confirm that, when a more intense broadband noise is presented to one ear, the change in the input–output functions in the other ear does not reduce the audibility of a softer speech sound presented to that ear, in such a way as to impair speech perception.

### Summary

Overall, modifications to speech-processing strategies, both when implemented commercially and when developed experimentally, have produced some small but inconsistent improvements. Whereas noise reduction and other pre-processing methods have consistently produced improvements of up to 25% correct or 2 dB reductions in SRT, gains from speech-processing strategies are typically less than 10% in terms of percent scores, and usually correspond to changes in SRT that are not larger than about 1–2 dB.

## FOCUSED AND CURRENT-SHARED STIMULATION

### Focused Stimulation

**Fig. 3:**
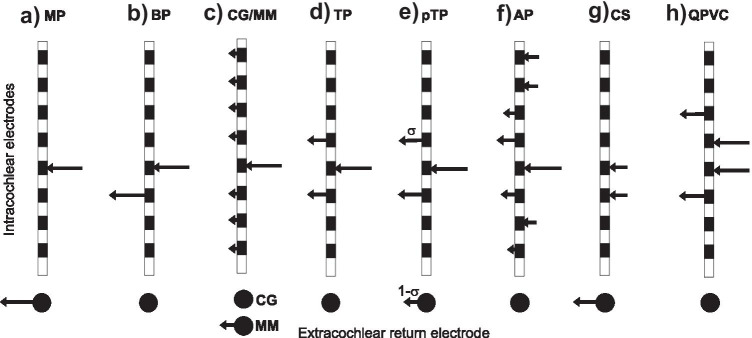
Schematic of the current pathway for different modes of stimulation: **a**) monopolar (MP), **b**) bipolar (BP), **c**) common ground (CG) and mixed mode (MM), **d**) tripolar (TP), **e**) partial tripolar (pTP), **f**) All-polar (AP), **g**) current steering (CS), and **h**) quadrupolar virtual channels (QPVC). The configuration in part (c) is mixed mode (MM) when a non-zero proportion of the injected current is, as shown, returned via the extra-cochlear electrode. When that proportion is zero, the configuration is equivalent to common ground

Regardless of the signal processing strategy used, twentieth century CIs usually presented electrical pulses in either monopolar (MP) or bipolar (BP) mode. As shown in Fig. 3a, b, this involves injecting current via one intra-cochlear electrode and returning it via either an extra-cochlear electrode in MP mode, or via a nearby intra-cochlear electrode in BP mode. Implants produced by Cochlear also permit common ground (CG) stimulation, in which current is returned equally among all non-stimulated intra-cochlear electrodes, and Oticon Medical exclusively uses a mixed mode (MM), which is a mixture of MP and CG (Fig. 3c). In all cases, it should be remembered that, although intra-cochlear electrodes are sometimes referred to as “active” and “return”, current will flow through all used intra-cochlear electrodes and can activate neurons close to those electrodes, and that the waveforms at active and return electrodes are simply inverted (and sometimes scaled) versions of each other.

A potential limitation imposed by MP stimulation arises from the broad current spread that it produces (Kral et al. 1998). As a result, although each electrode conveys information about only one frequency band, each neuron will respond to stimulation from many electrodes, each conveying information from a different input frequency band. Bipolar stimulation overcomes this problem to some degree, but has the drawback that both of the intra-cochlear electrodes will stimulate the auditory nerve, potentially leading to a bimodal excitation pattern, especially when the two electrodes in each pair are not immediately adjacent (see Fig. 3b; Kral et al. 1998; Macherey and Carlyon 2012a; Carlyon et al. 2017). Accordingly, researchers have investigated methods of producing more focused patterns of stimulation. One solution, that has been implemented experimentally in the Advanced Bionics device, is tripolar (TP) stimulation whereby current is injected via one electrode and returned by each of its two neighbours (Fig. 3d). An even more focused solution, in which, in principle, a non-zero voltage occurs at only one point along the electrode array, can be obtained using the “phased array” or “all-polar (AP) method (van den Honert and Kelsall, 2007). This is achieved by measuring the voltage spread from each electrode to every other electrode, expressing these measures as a matrix, and then inverting the matrix so as to obtain a non-zero voltage at only one location (Fig. 3f).

In principle, one might expect the reduced current spread produced by current-focusing methods to produce sharper neural excitation patterns and, possibly, improved speech perception. Indeed, some psychophysical experiments have reported reduced spread of excitation for tripolar compared to monopolar stimulation (Bierer and Faulkner 2010; Srinivasan et al. 2010; Fielden et al. 2013). However, other studies (Fielden et al. 2014; Marozeau et al. 2015) have failed to observe a difference, and, even when differences are observed, the reduction in spread of excitation with tripolar stimulation is usually modest and varies considerably across participants and across different electrodes in the same participant. One reason for this may be that, in order to obtain a sufficiently loud percept, the current level needs to be increased substantially for TP compared to MP stimulation. This has two consequences. First, the increase in current level may recruit neurons farther away from the stimulating electrode, thereby partially undoing the benefits of reduced current spread. For TP stimulation part of this recruitment may arise from “side lobes” introduced by the stimulation of the flanking (return) electrodes (Litvak et al. 2007). Second, in order to deliver a sufficiently high current given the compliance limits of the device, researchers usually have to use a “partial tripolar (pTP)” mode of stimulation in which a proportion σ of the injected current is returned via the intra-cochlear electrodes, with the remainder returned via an extra-cochlear electrode as in MP mode (Fig. 3e).

Studies that have implemented TP stimulation into signal processing strategies have also produced mixed results, with modest improvements obtained for speech in multi-talker babble in one single-blinded study (Srinivasan et al. 2013) and no benefit observed in other studies (Mens and Berenstein 2005; Bierer and Litvak 2016; Arenberg et al. 2018). A recent innovation is to dynamically vary σ as a function of input level so that the mode of stimulation is more similar to full tripolar at low levels and more similar to monopolar at high levels (Arenberg et al. 2018; de Jong et al. 2019). An unblinded study by Arenberg et al. (2018) found that this method improved the perception of vowels in four-talker babble (but not the identification of vowels in quiet or spondees in babble) compared to both MP and pTP strategies. In contrast, de Jong et al. {, 2019 #2068} found no benefit compared to an MP strategy for the identification of sentences in stationary noise, once they had controlled for learning effects.

A series of psychophysical experiments using AP stimuli showed results broadly similar to those obtained with TP stimulation: both reduced the electrical charge interactions between pulses presented to different electrodes, compared to MP stimulation, but did not reduce the spread of neural excitation (Marozeau et al. 2015). A limitation of the AP method is that, although it in principle restricts the spread of current to a single small region, the measurements used to obtain the solution are all obtained at the level of the electrode array rather than at the neurons. It is therefore likely that current will not be completely restricted to a single point along the auditory nerve array. Measurements from the cat inferior colliculus (IC), which, unlike the human psychophysical studies, show substantially reduced spread of excitation for TP compared to MP stimulation, also found that TP and AP stimuli produced very similar excitation patterns (George et al. 2015b).

### Current Steering

Another change to the mode of stimulation involves stimulating two adjacent electrodes with the same polarity and varying the relative proportion of the current delivered to the two electrodes (Fig. 3g). This “current steering (CS)” allows the generation of pitches intermediate to those generated by either electrode alone (Donaldson et al. 2005) although this can also be obtained to some extent by stimulating adjacent electrodes in quick succession rather than simultaneously (McDermott and McKay 1994), as happens anyway in standard CIS and n-of-m strategies. Current steering is implemented commercially in the HiRes120 and Optima signal processing strategies of Advanced Bionics. Both Donaldson et al. (2011) and Buechner et al. (2012) compared the HiRes strategy, which does not include current steering, to the HiRes120 strategy, which does. Neither reported any significant benefits for HiRes120. Current steering does however have the advantage of reducing power consumption (Frijns et al. 2009; Langner et al. 2017).

Landsberger and colleagues introduced the concept of combining current steering with current focusing, using the “steered quadrupolar” method (Landsberger and Srinivasan 2009). As shown in Fig. 3h, this involves sharing current between two adjacent same-polarity electrodes, as in regular current steering, but returning the current via two flanking electrodes with opposite polarity to the central ones. We are unaware of any study that evaluated speech perception with steered quadrupolar stimulation. A recent unblinded investigation of a related method (“steered tripolar”; Luo et al. 2020) found a small (1 dB) improvement in SRT for sentences in ten-talker babble, compared to an experimental monopolar strategy, with both strategies using a very low pulse rate.

### Summary

Focused stimulation methods can successfully reduce the current spread along the cochlea, at least when measured at the electrode array, and can substantially reduce the spread of neural excitation in animal studies. They can also reduce spread of excitation in humans, but the effects are modest and vary across patients and between electrodes in the same patient. Speech perception studies reveal either a small or no benefit for focused stimulation. Current steering can reduce power consumption but has not been shown to improve speech perception.

## PATIENT-SPECIFIC (BESPOKE) PROGRAMMING

The evidence reviewed in the “Signal Processing Strategies and New Stimulation Methods” and “Focused and Current-Shared Stimulation” sections suggests that advances in signal processing strategies and in novel modes of stimulation have produced, at best, modest and variable improvements in speech perception. A feature of both approaches is that they are “one size fits all”, with the same changes being implemented for all CI listeners. In fact, Skinner et al.’s (2002) comparison of the ACE, SPEAK, and CIS strategies reported a significant interaction between participant and strategy, providing (we think) the first statistical evidence for the potential benefits of patient-specific programming. However, rather than investigating methods for determining which signal processing strategy to assign to each patient, most research has concentrated on identifying electrodes, on a patient-by-patient basis, that should be deactivated (turned off). The underlying assumptions are that some electrodes evoke a less-faithful neural representation of the input signal, that listeners cannot ignore the neural response to these “bad” electrodes, and that therefore deactivating them will improve speech perception. Indeed, as discussed in the next section, there is good evidence that electrodes can differ strongly in the fidelity with which they convey information about the stimulus. Here we review the channel deactivation methods that have been used in an attempt to improve speech perception.

**Fig. 4:**
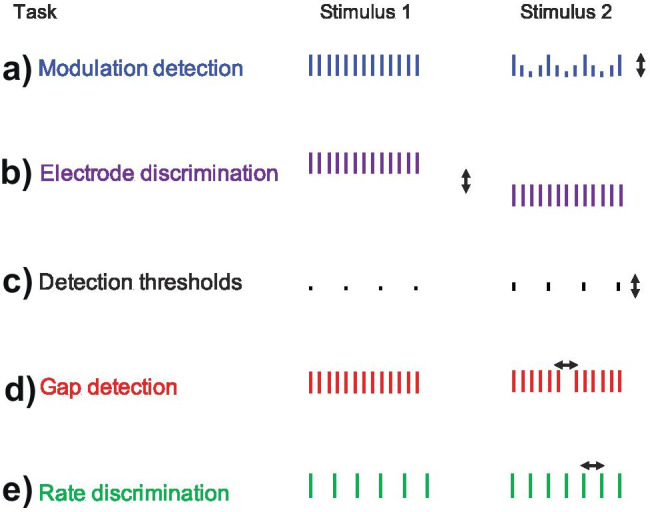
Left and right-hand columns show the standard and signal intervals of a 2IFC trial for various tasks that have been shown to vary substantially across electrodes. Solid lines in part **a**) illustrates the measurement of modulation detection thresholds (MDTs) in quiet; the dashed lines illustrate unmodulated pulse trains presented to an adjacent channel for the measurement of masked MDTs (Garadat et al. 2012). Other tasks are **b**) electrode discrimination, **c**) detection thresholds, **d**) gap detection, and **e**) rate discrimination

One of the first channel deactivation studies was reported by Garadat et al. (2012). They presented pulse trains to individual electrodes and, for each electrode, measured modulation detection thresholds (MDTs, Fig. 4a). This was achieved by modulating the duration of the biphasic pulses with a 10-Hz sinusoid and measuring the smallest amount of modulation that could be detected. Because the auditory nerve integrates charge over a duration of a few hundred microseconds, which is longer than the range of phase durations used in that study, the modulation of phase duration has a similar effect to amplitude modulation (AM). They also measured MDTs in the presence of an unmodulated masker presented to an adjacent electrode. They then created two 10-electrode versions of the CIS strategy, whereby the frequency-to-electrode map was modified to deactivate only electrodes having high or low masked modulation detection thresholds (MDTs). They reported better speech perception for the map with the high-MDT electrodes deactivated, with modest benefits of about 5% for vowels and consonants in noise and of 7% for sentences in quiet, with a 4 dB improvement in the SRT for sentences in stationary noise. A subsequent study, that compared the patients’ clinical (ACE) map to a version with 5 electrodes deactivated based on high-masked MDTs, reported mixed results, with improvements for sentence and consonant perception in noise but a decrement for vowel perception in noise (Garadat et al. 2013). In both studies, the pattern of masked and unmasked MDTs for each participant was highly correlated across electrodes, and so the deactivated channels would have been nearly identical if based on the unmasked MDTs. Hence, the critical feature of the deactivated channels appears to be related to the detection of modulation, rather than susceptibility to masking. Furthermore, because the modulation rate (10 Hz) was much slower than the value of about 100 Hz above which MDTs start to increase (Fraser and McKay 2012), it is likely that the limitation arises from amplitude processing rather than temporal acuity. Whatever the reason, interpretation of these significant and interesting results should be tempered by the observation that testing was not blinded in any way, so one cannot rule out the possibility that they were mediated by bias effects.

A second method is to deactivate electrodes that show poor electrode discrimination. Zwolan et al. (1997) tested Cochlear users of the now-discontinued “MPEAK” strategy and of a modified version of MPEAK in which electrodes were deactivated based on electrode discrimination performance. This unblinded study found benefits for some tests and participants at the individual level, but no overall benefit at the group level. Vickers (2016b) performed a randomised single-blinded crossover study with 13 users of the ACE strategy, comparing the clinical strategy to one with an average of 4 electrodes deactivated on the basis of poor discrimination. Participants were given at least 2 months take-home experience with the new map, but no benefits were observed for any of the speech measures used, including monosyllabic words and sentences masked by stationary noise or multi-talker babble. Furthermore, performance on the SMRT test (Aronoff and Landsberger 2013), which is a non-speech measure of spectro-temporal processing, was significantly worse with the experimental map. Another method that assessed the representation of pitch as a function of electrode position was used by Henshall and McKay (2001). They performed multi-dimensional scaling (MDS) of all electrodes of the Cochlear device, and deactivated electrodes where the results indicated that pitch might vary non-monotonically with electrode number. No benefits were observed for any of the measures tested, namely words and sentences in quiet and sentences in stationary noise.

A third method is to deactivate electrodes having high thresholds when stimulated in pTP mode. The rationale for this is based on computational evidence that high pTP thresholds can be caused by locally poor neural survival and/or large electrode-modiolar distance (EMD), both of which are likely to produce broad neural excitation patterns (Litvak et al. 2007; Goldwyn et al. 2010; Kalkman et al. 2015). Bierer and Litvak (2016) deactivated between 1 and 6 electrodes of the Advanced Bionics device that had high thresholds, but this did not produce an overall improvement in either vowel or consonant identification.

A fourth approach has been to deactivate electrodes having high thresholds for low-rate pulse trains presented in monopolar mode. Zhou (2016) showed that, for a given listener, forward-masked excitation patterns are broader for electrodes giving high detection thresholds for 80 pps pulse trains than for electrodes giving low thresholds. It was therefore argued that these thresholds could be used as a simple estimate of the width of the neural excitation pattern of each electrode. Zhou tested 10 Cochlear participants comparing their clinical map to an experimental map in which five electrodes with high 80 pps thresholds were deactivated. SRTs for sentences in modulated noise and percent-correct scores for sentences in quiet were both better for the experimental map, with effect sizes of 4 dB and 11%, respectively (Zhou 2016). Broadly similar improvements were observed in a subsequent study where participants were given take-home experience with the experimental map. The improvements were similar for different masker types, including steady noise and an interfering talker. Zhou (2017) also showed that performance on a non-speech test of spectro-temporal processing (“SMRT”: Aronoff and Landsberger 2013) was better for the experimental than for the clinical map. Furthermore, the difference in SMRT scores between the two maps was correlated across participants with the difference in the SRT for sentences in modulated noise. The differences between the experimental and clinical maps observed in all three studies are non-trivial, with the SRT improvements of 3–4 dB comparing favourably with those obtained by noise reduction methods (the “Pre-processing Strategies” section). A *caveat* is that all three studies used a single-blinded rather than a double-blinded design, so one cannot completely rule out the possibility of experimenter effects. This may be more of an issue for speech tests, especially for the measurement of the SRT, where the SNR is controlled by the experimenter based on their scoring of each verbal response, but is likely to be less of an issue for tests such as the SMRT where the scoring and stimulus control are completely automatic.

A fifth method has been to use modelling based on post-operative CT scans to identify and deactivate electrodes that are likely to produce broad or distorted excitation patterns—for example those located far from the location of spiral ganglion neurons in the modiolus. Noble et al. (2014) used this approach in a large study involving 68 participants, some of whom were bilaterally implanted, and reported small but significant average differences between the experimental and clinical maps. Testing with the clinical map always occurred first, followed by 3–6 weeks’ experience with the experimental map. Unfortunately, this design is susceptible to practice effects, such that the improvement might for example be due to increased familiarity with the test materials (Psarros et al. 2002; Vermeire et al. 2010; de Jong et al. 2019). Indeed, the differences between test sessions observed with a smaller number of tested ears, whose implants were not re-programmed, overlapped substantially with those having deactivated electrodes. These issues also apply to two later studies (Labadie et al. 2016; Danieli et al. 2021). This does not of course mean that CT scans are never useful for electrode deactivation; for example, extreme distortion of the electrode array, such as tip foldover, will likely have a severe impact on the representation of the auditory stimulus and will warrant deactivation (Danieli et al. 2021). However, it does mean that we do not yet have strong evidence supporting widespread and systematic use of CT-based channel deactivation strategies.

Finally, we have tested a strategy that deactivates neurons based on the effect of the polarity of asymmetric pulses on detection thresholds (Goehring et al. 2019b). The strategy was motivated by evidence, discussed in the next section, that this “polarity effect (PE)” may reflect local neural health. At group level, no significant differences for sentence identification, either in time-reversed speech noise or in quiet, were observed between two maps in which 5 out of 15 electrodes were deactivated based on large vs. small polarity effects, or between these experimental maps and a third map in which no electrodes were deactivated. The experimental maps also did not affect overall performance at the group level for a non-speech test that measured spectro-temporal processing (“STRIPES”: Archer-Boyd et al. 2018), although there was a significant correlation such that the STRIPES test could, to some degree, predict which strategy produced better speech performance on a listener-by-listener basis.

**Fig. 5:**
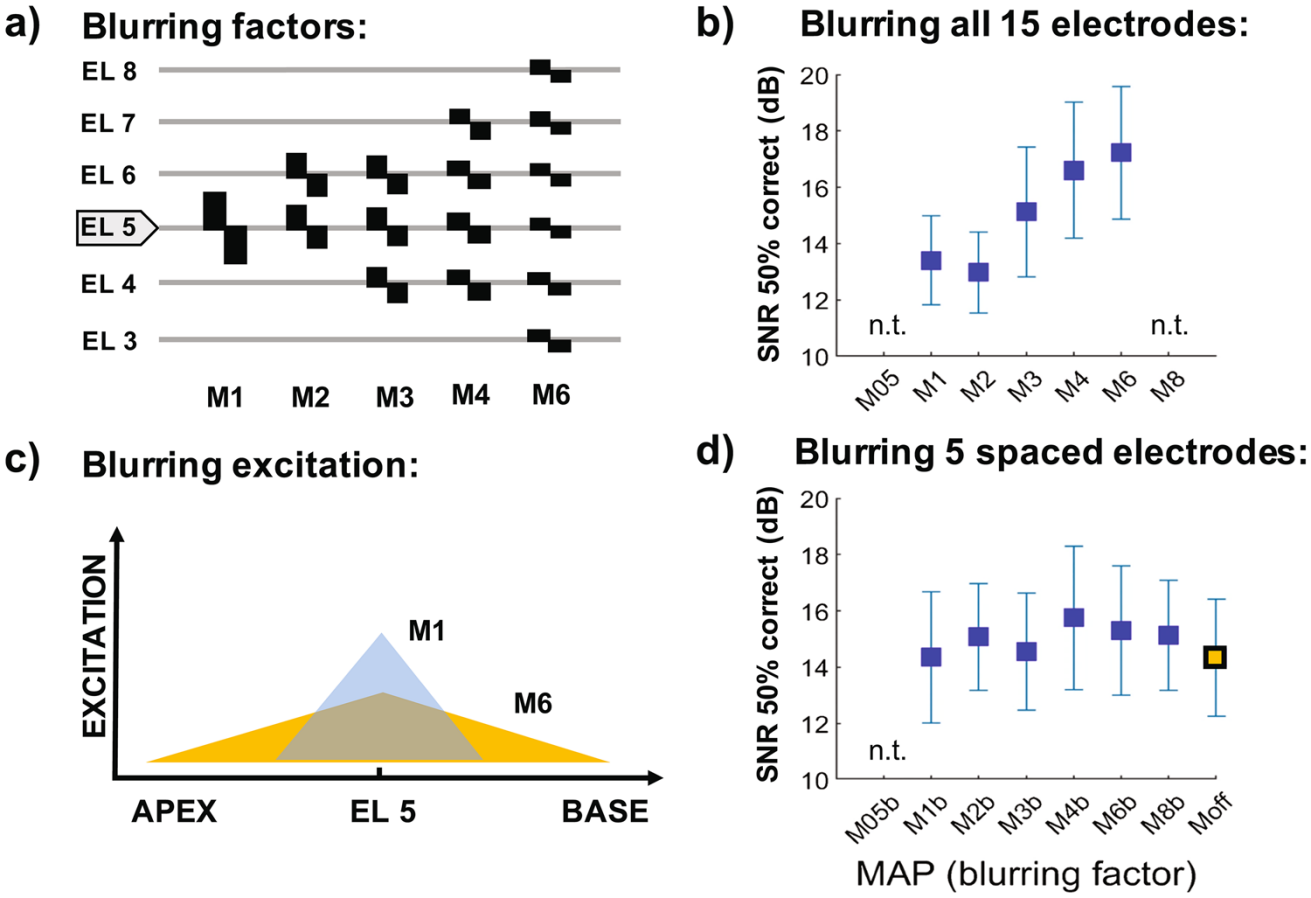
**a**) Illustration of the spectral blurring imposed by Goehring et al. (2020; see text) and for stimulation of channel 5. Condition M1 is the standard condition where a single electrode is stimulated in monopolar mode. Conditions M2, M3, M4, and M6 represent increasing amounts of blurring, whereby multiple adjacent electrodes are stimulated simultaneously. Parts **b**) and **d**) show the SRTs obtained when blurring all 15 or 5 evenly spaced electrodes, respectively. Part **c**) illustrates the point that a narror excitation produces more excitation at its peak than is the case for a broader but equally loud excitation pattern

Three of the metrics described above—low-rate thresholds, electrode discrimination, and CT-imaging models—aim to deactivate electrodes that produce broad spreads of neural excitation. In addition, most studies incorporate an additional rule that avoids deactivating groups of adjacent electrodes and instead distributes the deactivated electrodes more evenly across the array. We recently investigated how effective such an approach is likely to be in principle, even if one could identify a small number of electrodes that produced very wide excitation patterns (Goehring et al. 2020, 2021). Instead of conveying each channel using one electrode as in standard strategies, we simulated wide excitation patterns by simultaneously stimulating a number of adjacent electrodes based on the output from one filter (Fig. 5a). As expected, applying this “blurring” to all channels impaired identification of sentences masked by time-reversed speech (Fig. 5b). However, no deficit was observed when five evenly spaced electrodes were blurred, even for the most extreme case where each channel was conveyed by stimulation of eight adjacent electrodes (Fig. 5d). Deactivating the blurred electrodes also had no effect. One reason for this—which we believe also applies to cases where single-electrode stimulation produces a broad excitation pattern—is that each channel in a speech-processing map is adjusted to produce approximately the same loudness. Consequently, a broad excitation pattern will have a lower excitation than a sharp pattern at places along the auditory nerve array that are far from the stimulating electrode (Carlyon et al. 2017), thereby reducing its ability to mask neighbouring electrodes with sharp excitation patterns (Fig. 5c). We therefore believe that deactivating a modest number of evenly spaced electrodes on the basis of their broad excitation patterns is a priori unlikely to improve speech perception. It is possible, however, that more severe local distortions of the excitation pattern related to neural function, for example those produced by so-called neural dead regions, would degrade speech perception and might be alleviated by electrode deactivation. Shannon et al. (2002) simulated some consequences of neural dead regions by setting the stimulation levels on sets of adjacent electrodes to below detection threshold, and found that applying this manipulation to a sufficiently large number of electrodes—usually 6 or more—could impair speech perception. Their simulation meant that the corresponding parts of the speech spectrum were not presented to the listener, as is the case for a real dead region, but differed in that neurons in the simulated dead region would have responded to adjacent frequency bands rather than being absent. One finding that is consistent between the “blurring” and “dead region” simulations is that speech scores can be degraded only by applying a manipulation to several adjacent electrodes, which is different from the approach commonly adopted by channel deactivation strategies that typically avoided deactivating clustered groups of electrodes.

### Summary

Efforts for bespoke programming strategies have led to mixed results and only very few studies have shown clear, but modest improvements. For strategies that aim to improve speech perception by deactivating subsets of electrodes in regions of bad neural function, it remains unclear which measure to use to best identify such electrodes and whether different measures would lead to similar benefits in speech perception (see also Brochier et al. 2021). Furthermore, studies that simulated wide or distorted excitation patterns only led to significant deteriorations of speech perception when several adjacent electrodes were affected concurrently. Hence, strategies that aim to improve speech perception by deactivating subsets of electrodes with broad excitation patterns are unlikely to succeed by deactivating individual electrodes that are spaced out along the array. This conclusion is based on the rationale that if broadening the excitation pattern produced by an electrode does not decrease performance in the first place, then deactivating that electrode is unlikely to help.

## THEORETICAL AND SCIENTIFIC ADVANCES RESULTING FROM CI RESEARCH

As with any medical device, progress in the study of CIs should not be assessed solely in terms of immediate clinical benefits. Rather, advances in our understanding of the auditory system’s response to electrical stimulation are of scientific value and pave the way for future developments that may improve patient health. Although we should not underestimate the role of multiple incremental advances in our quantitative understanding of the response to CI stimulation, here we focus on what we have termed in the Introduction “reliable surprises”. These are defined as findings that would not have been expected based on our knowledge at the turn of the century and that have been replicated, preferably in more than one laboratory.

### Polarity Effect

**Fig. 6:**
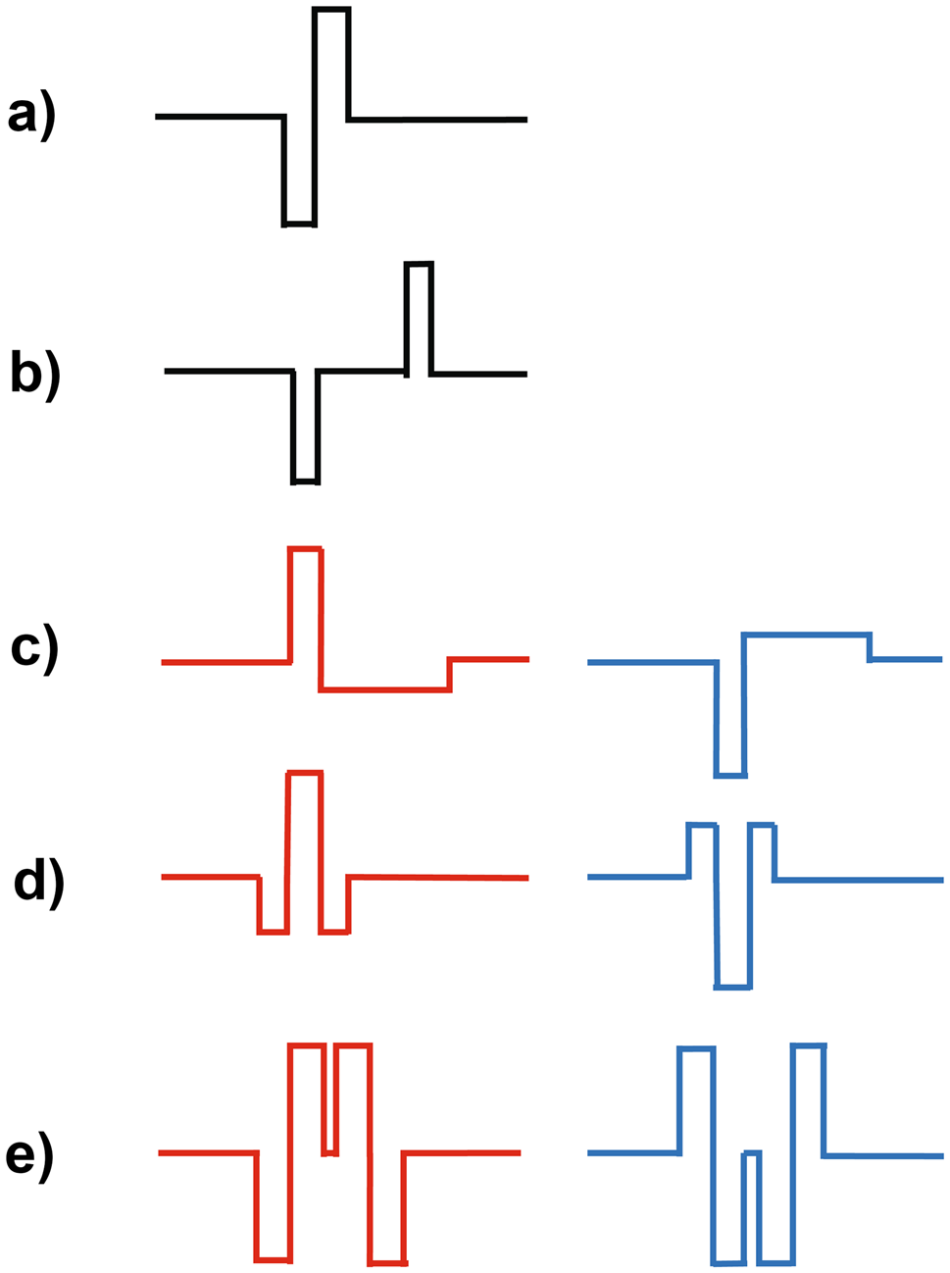
Parts **a**) and **b**) show a symmetric biphasic pulse with short and long inter-phase gap, respectively. Parts **c**), **d**), and **e**) each show an asymmetric pulse shape with either the anodic (red, left column) or cathodic (blue, right column) charge concentrated into a short time period. Pulse shapes are **c**) pseudomonophasic, **d**) triphasic, and **e**) quadraphasic

In clinical use, most CIs present symmetric biphasic pulses (Fig. 6a, b). This satisfies the safety requirement for charge-balanced stimulation, but makes it hard to determine which phase most effectively stimulates the auditory nerve. Experiments using monophasic pulses presented to cats and guinea pigs showed that the cathodic phase was the most effective (Hartmann et al. 1984; Miller et al. 1999a, b). However, Macherey et al. (2006) found that the opposite was true for humans, for whom anodic stimulation was most effective. To demonstrate this, they used so-called pseudomonophasic pulses—charge-balanced pulses consisting of a short high-amplitude phase followed by a longer low-amplitude phase of the opposite polarity (Fig. 6c). Macherey et al. (2006) found that the current needed to reach most comfortable loudness (“MCL”) was lower when the high-amplitude phase was anodic than when it was cathodic. It has subsequently been shown that the same direction of polarity effect (“PE”) can be obtained using different types of asymmetric pulse (Fig. 6d, e; Carlyon et al. 2013). For brevity, we refer to pulse shapes where the anodic/cathodic current is focused into a short time period as “anodic” and “cathodic” stimuli, respectively. The direction of the PE—greater sensitivity to anodic than to cathodic stimulation—has been confirmed psychophysically using loudness adjustment (Carlyon et al. 2013), masking (Macherey et al. 2008, 2010), and pitch perception (Macherey et al. 2011; Macherey and Carlyon 2012b) measurements. It has also been demonstrated using the electrically evoked compound action potential (ECAP: Macherey et al. 2008; Undurraga et al. 2010; Spitzert and Hughes 2017) and the electrically evoked auditory brain response (Undurraga et al. 2013). The size of the effect is on average about 2 dB, which corresponds to a substantial proportion (approximately 25–35%) of the typical electrical dynamic range with CIs. A clinical application of the finding has been realised in the MedEl device, where it has been shown that anodic-triphasic pulses can be used to reach MCL without causing unwanted stimulation of the facial nerve (FN: Bahmer and Baumann 2016; Bahmer et al. 2017). FN stimulation is a side effect of CI stimulation that occurs in about 5–6% of cases, although estimates of its occurrence vary somewhat across studies (Van Horn et al. 2020). As a result, the use of the anodic-triphasic pulse shape is now recommended for patients with excessive facial nerve stimulation (MedEl 2018). Anodic-pseudomonophasic pulses are also the default pulse shape used in CIs produced by Oticon Medical.

The most likely explanation for the PE, and for its difference in sign between humans and animals, rests in between-species differences in the anatomy and possibly status of the auditory nerve. Modelling studies show that cathodic pulses preferentially stimulate the peripheral processes of SGNs, while anodic pulses preferentially stimulate the central axon (Rattay et al. 2001; Joshi et al. 2017; Resnick et al. 2018; Potrusil et al. 2020). The peripheral processes may have degenerated in human CI listeners as a result of months or years of auditory deprivation (Johnsson et al. 1981), therefore leading to greater sensitivity to anodic stimuli. In contrast, the subjects used in most animal experiments are acutely deafened, leading to preserved peripheral processes and greater sensitivity to cathodic stimulation. However, for guinea pigs, there is preliminary evidence that the sign of the PE does not depend on duration of deafness, suggesting that factors other than degeneration of the peripheral processes are important (Macherey and Cazals 2016). These factors are likely related to modelling evidence suggesting that the PE may depend on the electrode-modiolar distance and on anatomical features of the peripheral auditory system such as the orientation of auditory nerve fibres relative to the stimulating electrode (Rattay et al. 2001).

Although the PE is sizeable and consistent across participants and electrodes for stimuli presented at MCL, at threshold the effect is smaller and its direction can differ both across participants and between electrodes within the same CI (Macherey et al. 2017; Carlyon et al. 2018a; Jahn and Arenberg 2019; Mesnildrey et al. 2020). One possible reason for this is that fewer neurons are required to fire at threshold than at MCL, and that, for some electrodes, there might be sufficient nearby peripheral processes remaining, leading to greater sensitivity to cathodic pulses. It has therefore been suggested that the size and direction of the PE at threshold may serve as an index of the survival of the peripheral processes of the auditory nerve. Evidence consistent with this idea comes from three studies showing that electrodes that have lower overall thresholds tend to produce lower thresholds for cathodic than for anodic stimuli (Carlyon et al. 2018a; Jahn and Arenberg 2019; Mesnildrey et al. 2020).

### Across-Electrode Variations in the Electrode-Neuron Interface

The interest in channel deactivation methods described in the “Patient-Specific (Bespoke) Programming” section stems from a number of findings that, we believe, collectively qualify as a “reliable surprise”. They show that the response of the auditory system to stimulation of a single electrode varies idiosyncratically, reliably, and sometimes markedly across the different electrodes in a single CI recipient. This is true even for monopolar stimulation, which produces a broad current spread, and where the excitation patterns produced by nearby electrodes are expected to overlap substantially. Some of these measures have been used in channel deactivation studies and have been described in the “Patient-Specific (Bespoke) Programming” section. These include masked and unmasked MDTs (Fig. 4a; Garadat et al. 2012, 2013), electrode discrimination (Fig. 4b; Zwolan et al. 1997; Vickers et al. 2016b), detection thresholds (Fig. 4c; Bierer et al. 2010, 2015; Zhou 2016), and, as also discussed above, the PE at threshold (Carlyon et al. 2018a; Jahn and Arenberg 2019; Mesnildrey et al. 2020). As noted in the “Patient-Specific (Bespoke) Programming” section, we believe that the across-electrode variation in MDTs primarily reflects amplitude coding, that detection-threshold measures may reflect differences in the spread of excitation and/or neural survival, and that the PE may reflect the survival of the peripheral processes of the auditory nerve.

The presence of across-electrode differences is not limited to effects that have been used to guide channel deactivation studies. For example, reliable across-electrode differences have also been observed for gap detection (Fig, 4d: Garadat and Pfingst 2011; Bierer et al. 2015) and pulse-rate discrimination, at both low and high pulse rates (Fig. 4e; Carlyon and Deeks 2015; Cosentino et al. 2016). Furthermore, between-electrode differences in humans have been interpreted using evidence from animal studies that have combined psychophysical or electrophysiological methods with histological measures of the extent of neural survival (Pfingst et al. 2015). One of these is multi-pulse integration (MPI), defined as the slope of the function relating threshold to pulse rate, and which has been shown to be correlated with neural survival in guinea pigs (Zhou et al. 2015; Zhou and Pfingst 2016). Another effect concerns across-electrode differences in the dependence of the ECAP on the duration of the gap that separates the two opposite polarity phases of a biphasic pulse (Fig. 6a vs. 6b: Schvartz-Leyzac and Pfingst 2016; Brochier et al. 2021). The effect of this inter-phase gap (IPG) on the ECAP was previously shown to be positively correlated with neural survival in guinea pigs (Prado-Guitierrez et al. 2006; Ramekers et al. 2014). Significant across-electrode differences have also been observed in EEG measures including the electrically evoked auditory steady-state response (Gransier et al. 2020) and the neural distortion response (Carlyon et al. 2021), both of which reflect phase-locked neural activity in the auditory thalamus and/or cortex. Mathew et al. (2017) reported a cortical analogue of electrode discrimination (Fig. 4d), termed the electrically evoked auditory change complex, and showed that it correlated somewhat with behavioural measures of electrode discrimination.

Here we consider two important and related issues pertaining to the across-electrode variations observed in the different measures. One of these concerns the possible neural bases for the variations, which a number of studies have attributed to across-electrode differences in local “neural survival” or “neural health” (e.g. Bierer 2007; Pfingst et al. 2011, 2015; Bierer et al. 2015; Cosentino et al. 2016; Zhou and Pfingst 2016; Schvartz-Leyzac et al. 2020). It would be helpful to have a more specific hypothesis of how a particular aspect of neural health or survival should affect each psychophysical or electrophysiological measure. The second issue concerns the extent to which the across-electrode variations are correlated across measures. Clearly, if across-electrode variation were driven either by a single factor, for example the number of surviving neurons, or by several highly correlated factors, then the different measures should be correlated highly with each other. Conversely, if there were multiple factors that varied more or less independently across the electrode array, then the different measures might not be correlated. Both issues were recently addressed in a study by Brochier et al. (2021), who measured across-electrode variation in MPI, in the polarity effect (PE), and in the effect of IPG on ECAPs for a group of 11 CI users. They found that although each measure varied reliably and idiosyncratically across the electrode array, the variations in the three measures were not correlated with each other. They then passed the stimuli through a phenomenological spiral ganglion model (Joshi et al. 2017) to determine which aspects of neural health might account for across-electrode variations in each measure. The results suggested that the IPG effect was likely to be most affected by central axon demyelination, the polarity effect was likely dominated by the survival of the peripheral processes, and the MPI should be greatest when the standard deviation of the thresholds of the different neurons responding to the stimulus is large. They noted that electrodes with larger EMDs might produce broader excitation patterns and a large across-neuron threshold standard deviation, consistent with psychophysical evidence that electrodes that produce larger MPIs also produce broader excitation patterns (Zhou and Pfingst 2016).

Several other studies have revealed differences in the extent to which the various measures are correlated with each other*.* Pfingst (2015) described data from two participants for whom the across-electrode variations in MDTs, MPI, gap detection, and MCLs differed markedly between the different measures. Consistent with Brochier et al.’s (2021) results, Schvartz-Leyzac et al. (2020) found no correlation between MPI and the IPG effect. Bierer et al. (2015) found that across-electrode variations in gap detection and in detection thresholds were both correlated between MP and pTP stimulation, but that the two measures were not correlated with each other. On the other hand, there is evidence that electrodes that produce broader excitation patterns exhibit more MPI (Zhou 2016) and higher thresholds for low-rate pulse trains (Zhou and Pfingst 2016), and that the PE at threshold is correlated with the threshold when averaged across polarities or measured with symmetric pulse shapes (Mesnildrey et al. 2017; Carlyon et al. 2018a; Jahn and Arenberg 2019). Furthermore, Cosentino et al. (2016) reported that the “upper limit of temporal pitch”—the pulse rate above which pitch does not increase—was significantly correlated across electrodes with gap detection thresholds but not with pulse-rate discrimination limens at low rates. Collectively, these findings go some way towards the goal of identifying clusters of tests whose results are correlated with each other but not with the results of other tests, and that might share a common biological basis. Further information on between-test correlations might allow computational models to provide basic insights into what those biological bases might be, and would in turn constrain the models to account for the different inter-test correlations. This may also be of some practical value, by allowing one to determine which clusters of tests could most effectively inform channel deactivation methods (cf. the “Patient-Specific (Bespoke) Programming” section), and provide a stronger theoretical basis for using those methods.

### Effects of Long-Term Deprivation and Restored Auditory Stimulation

CIs provide an almost unique opportunity to study the effects of both long-term deprivation and, importantly, subsequent restoration of hearing on auditory processing. Some evidence for auditory plasticity was available at the turn of the century, for example on the effects of CIs on cortical maturation in deaf children (Ponton et al. 1996). Subsequently Hughes (2001) reported a comprehensive study describing the increases in MCL that occur for both adults and children in the months following the initial activation of a CI. Since then, there has been considerable progress in identifying changes, not only in sensitivity but also in the tonotopic selectivity and temporal encoding of the neural response to electrical stimulation. Experiments with cats before the turn of the century had revealed that long-term auditory deprivation following neonatal deafening substantially degrades the cochleotopic representation in auditory cortex (Raggio and Schreiner 1999). Fallon and colleagues (Fallon et al. 2009, 2014a) have more recently shown that this degradation is small or absent after chronic electrical stimulation in kittenhood, and that it can even be restored, to some extent and in a subset of animals, by chronic stimulation starting in adulthood. Interestingly, long-term deprivation appears to have a significant but milder impact on neural excitation patterns measured in the IC than in the cortex; tonotopy is maintained but excitation patterns become broader (Vollmer et al. 2007; George et al. 2015a). Further evidence for the restoration of auditory processing post CI implantation comes from a study with ferrets (Isaiah et al. 2014), that showed lower performance for the earlier deafened group in an auditory localization task with bilateral CIs, but observed significant training-induced improvements with interleaved auditory and visual stimuli in line with multisensory integration and cross-modal reorganisation.

Studies completed in this century have also revealed plasticity in temporal processing in response to electrical stimulation. One line of evidence comes from the earlier finding that neurons in the cat IC phase lock to electric pulse trains only for pulse rates up to a certain value (Snyder et al. 1995). This “upper limit” has been shown to be higher for cats that have grown up either with normal hearing or listening through a CI than for those that have grown up deaf and with no electrical stimulation (Hancock et al. 2013; Vollmer et al. 2017). Rabbits who have grown up with normal hearing also show superior temporal processing in the IC compared to those deafened at birth, although in this case the difference is primarily in the number of neurons that phase lock to electrical stimulation rather than in the upper limit (Chung et al. 2019). Interestingly, there is evidence for plasticity at the level of the brainstem even in adulthood. Vollmer et al. (2005) reported that chronic stimulation as an adult can restore the upper limit of phase locking in the IC of cats that have been deaf since birth. When measured in the IC, the upper limit is also higher for acutely deafened cats than for cats that have been deafened as adults 6 months before the measurements (Middlebrooks 2018). Phase locking of cells in the auditory cortex is also affected by the history of stimulation and by training (Vollmer and Beitel 2011; Fallon et al. 2014b; Vollmer et al. 2017). In a behavioural experiment, Carlyon et al. (2018b) investigated whether, analogous to the physiological findings of Vollmer et al. (2005), human CI listeners would show an increase in the psychophysical upper limit of temporal pitch during the months following the initial activation of their device. They measured pitch ranking of single-electrode pulse trains on the day the CI was first activated and at 2, 6, and 9 months later. The upper limit did indeed increase, with the largest change occurring during the first 2 months. Improvements in behavioural measures may arise from increased familiarity with the test materials, and so it is important to differentiate between this “procedural” learning and stimulation-induced plasticity. Carlyon et al. (2018b) noted that the increase in upper limit was greater between than within sessions, had a larger effect size than the improvement in a low-rate pitch task, and that a previous study of long-term CI users had shown no increase in upper limit across multiple sessions. These considerations reduce but do not eliminate the possibility that the results were affected by learning. In addition, because stimuli were always presented at MCL, which increased over time, it was hard to disentangle the effects of experience and stimulus level. As Carlyon et al. (2018b) pointed out, a similar *caveat* applies to single-unit electrophysiological measures, for which the stimulus level is typically adjusted so as to produce a similar overall response rate in each animal tested. Long-term CI stimulation might increase the current needed to elicit a response, and the higher upper limit compared to un-stimulated animals might reflect the increased stimulus current rather than changes in temporal processing per se. Another study of experience-dependent changes in temporal pitch perception by human CI users was performed by Goldsworthy and Shannon (2011) who argued that because the pitch percept at high pulse rates is weak, it might be especially susceptible to focussed training methods. They found that pulse-rate discrimination could be improved by extensive training. However, because this improvement was not greater at high rates than at low rates, and because it was observed using test stimuli that were similar to those used for training, we believe their results are more consistent with a general (e.g. procedural) learning or training effect rather than to any training-induced sensory plasticity that is specific to high-rate processing.

Further evidence on auditory plasticity arises from the fact that CIs are being increasingly provided to patients who have significant remaining acoustic hearing in the implanted or unimplanted ear. This has allowed researchers to estimate the pitch produced by stimulation of individual electrodes by asking participants to compare it to the pitch produced by acoustic stimuli. A number of studies have reported that these pitch measurements can change in the months following implantation, and have suggested that these changes can be attributed to the combined acoustic and electric stimulation that patients experience in everyday life (Reiss et al. 2007, 2008). One *caveat* is that the pitch comparisons made in such studies are highly susceptible to non-sensory bias effects, such as may arise from the range of acoustic stimuli used for each pitch comparison (Carlyon et al. 2010; Schatzer et al. 2014; Goupell et al. 2019).

The need to distinguish between the possible bases for longitudinal changes in CI performance is especially pertinent to studies of speech perception, which are necessarily measured using behavioural techniques. A number of studies this century have examined the improvement that occurs during the months following implantation, following changes in the frequency-to-electrode map, or as a result of training regimes (Fu et al. 2002, 2005; Fu and Galvin 2008; Holden et al. 2013). This section has so far considered two very different ways in which speech scores might increase over time, namely improvements in tonotopic and/or temporal sensory coding vs. increased familiarity with the test materials and/or procedures (for a review of different types of learning in normal-hearing (NH) listeners, see Ortiz and Wright 2009). Improvements in speech scores may also arise from an increased ability to map the new or changed sensory representation produced by a CI onto the representations of speech sounds. This type of learning was investigated in a series of studies in which NH listeners were trained to understand noise-vocoded speech, and which showed that the improvement generalised to novel words (Hervais-Adelman et al. 2008). Generalisation between test and training materials has also been observed for CI listeners, including a series of studies summarised by Fu and Galvin (2008), and in which procedural learning was minimised by testing the participants on multiple occasions prior to the start of the experiment. For example, Fu et al. (2005) trained 10 poor-to-moderate CI users on the discrimination of monosyllables using an adaptive procedure for 1 h per day, 5 days per week, for a month. Listeners were tested before and after training using different materials, namely the forced-choice identification of vowels or consonants produced by multiple talkers who differed from those in the training set. Improvements of 14% and 16% were observed in both tests, and three listeners who additionally performed a sentence test showed substantial improvements. Hence, it appears that training can improve speech perception scores in CI listeners, that it can generalise somewhat to non-trained stimuli, and that these improvements are unlikely to be solely attributable to procedural learning. One *caveat* is that it is challenging to design a suitable placebo control for training (or other longitudinal) studies, and most studies do not do so. This could be achieved either by including a task that is definitely not predicted to improve as a result of training, or to include a control training regime that is plausible but expected to be less effective.

Evidence has also emerged for cross-modal, rather than purely auditory, plasticity in CI users following activation of their device. Several studies using measures such as magnetic resonance imaging (MRI), electroencephalograpy (EEG), and functional near-infrared spectroscopy (fNIRS) investigated effects of cross-modal activity induced by auditory and visual stimulation (Finney et al. 2001; Lomber et al. 2010) and their association with speech perception outcomes with CIs. Some studies have reported that listeners with stronger activation of auditory cortical areas by visual stimulation had worse speech outcomes, which was attributed to a maladaptive mechanism that led to a “visual take-over” of auditory areas due to long-term deprivation (Lee et al. 2001, 2007; Doucet et al. 2006; Sandmann et al. 2012). However, other studies measured activity in auditory regions to visual speech before CI implantation and found an opposite, positive relationship with CI performance 6 months after implantation (Anderson et al. 2017), which was attributed to adaptive cross-modal benefits for more efficient audio-visual integration after CI implantation (Rouger et al. 2012). It has also been shown that activity in visual or audio-visual cortical areas during a speech listening task performed after CI implantation was a significant positive predictor of CI performance on an audio-visual speech test 6 months later (Strelnikov et al. 2013). This mixed picture may have arisen from differences between studies in terms of the physiological and behavioural methods used, the time points of the activity measurements (before/after CI implantation), and onset of language development in the participants (pre/post-lingual). In addition, the correlational nature of these studies, combined with the possible existence of both within- and between-subject biases, hampers the identification of the underlying mechanisms and the direction of causality. For example, a negative correlation between visual activation of auditory cortices and auditory-only speech perception might reflect maladaptive plasticity, or alternatively be due to speech perception being impaired by sub-cortical degradation of the auditory system, leading to cortical resources being (adaptively) re-assigned to visual processing. This distinction is important because in the first example visual activation is a “bad” thing, being the cause of poor speech perception, whereas in the second it is a “good” thing in that it helps patients with poor sub-cortical processing to understand speech. The distinction may also have clinical implications, for example in determining whether therapies that involve access to visual speech will do harm or good.

The issue of cortical plasticity is of particular importance for the growing numbers of infants and children undergoing cochlear implantation. For example, the trajectories of cortical plasticity with CIs may differ somewhat between CI listeners who have had auditory input and language acquisition before deafness and subsequent CI provision compared to those who did not (Petersen et al. 2013). Furthermore, differences in neurodevelopmental plasticity between the juvenile and the mature auditory system can lead to different trajectories with age (Kral, 2013). Related to this, the existence of sensitive periods and time limits for successful cortical plasticity in congenitally deaf children provides evidence that early CI provision (best before the second year of life) is important to maximise outcome potential and that delaying implantation until the age of 8 or over can have a negative effect (Harrison et al. 2005; Kral and Sharma 2012; Kral 2013; Kral and Sato 2020). Late implantation led to abnormal audio-visual integration in the McGurk test (Schorr et al. 2005), with a dominance of the visual stimulus for children with CIs in comparison to a dominance of the auditory stimulus for normally hearing children in cases with conflicting audio-visual stimuli. Some of the children with early-implanted CIs exhibited strong bimodal fusion that was indistinguishable from that for normal-hearing children with strong bimodal fusion. However, the likelihood of this positive outcome was reduced with increasing age at implantation, in line with reports of a sensitive period for early implantation that leads to better speech and language outcomes.

### Summary

Taken together, new evidence for the effects of acoustic stimulation on preventing the loss of and/or restoring effective processing of electrical stimulation may provide important insights into the neural basis of auditory plasticity. In addition to enhancing our understanding of the operation of the auditory system, these findings may shed light on why speech perception is affected by long-term deafness and why it typically improves in the months following implantation. Although the effects of experience on speech perception are undoubtedly influenced by high-level processes, such as patients learning to map the novel sensations produced by a CI onto learnt representations of speech segments (Davis et al. 2005), plastic changes in basic sensory processes may well play a part. An improved understanding of plasticity along the auditory pathway after CI provision and of the adaptive mechanisms at play also has the potential to improve outcomes. For example, it would be useful to know whether to recommend auditory-visual or auditory-only training to new CI listeners, and whether the appropriate form of rehabilitation differs between those with pre-lingual and post-lingual onset of deafness.

## DISCUSSION

### How Well Have We Done?

The twenty-first century developments described in the “Pre-processing Strategies”, “Signal Processing Strategies and New Stimulation Methods”, “Focused and Current-Shared Stimulation”, and “Patient-Specific (Bespoke) Programming” sections have produced varying degrees of success in improving hearing by CI listeners by means of processing and stimulation strategies. The introduction of directional microphones has produced substantial improvements of speech perception in the presence of spatially separated noise, and noise reduction algorithms have produced more modest but robust benefits even when speech and noise are co-located. Arguably, however, these improvements have been achieved by applying technology developed from research on hearing aids and on signal processing. New CI speech-processing strategies have produced moderate improvements in some experiments, although a clear “winner” that produces substantial improvements in a wide range of studies and listening situations is yet to emerge. Similarly, methods for reducing current spread by using focussed stimulation have achieved modest but variable success, and most manufacturers still use MP mode as the default or only method of stimulation. Some benefits have also been realised by methods that deactivate “bad” channels in a patient-specific manner, inspired by the substantial and idiosyncratic across-electrode variations that can be observed in psychophysical and electrophysiological measures. However, no uniformly accepted method has yet been adopted into widespread clinical use, and the evidence for programming criteria to guide channel deactivation clinical choices is still emerging.

In some ways, the present CI landscape differs markedly from that at the turn of the century. In terms of basic research, much more is now known about the effects of neural plasticity and about the way in which the human auditory nerve (AN) is driven by electrical stimulation, and there is increased awareness of the idiosyncratic differences in the effectiveness of stimulation between different CI electrodes. Clinically, nearly all patients now use devices worn behind the ear or on the head and that incorporate directional microphones and effective noise-cancellation techniques. All modern CIs incorporate back telemetry, allowing the clinician to verify the AN response to electric stimulation. Arguably the most striking change, though, has been the marked expansion of the availability and applications of CIs: more and younger children receive implants, binaural implantation is commonplace, and implantation criteria have been relaxed to include increasing numbers of people with residual acoustic hearing in the implanted or unimplanted ear (Vickers et al. 2016a). It is interesting to consider the extent to which these advances owe their success to the substantial research effort that has taken place over the last 21 years. In some cases, the link is clear. For example, the effective combination of acoustic and electric hearing in the same ear depends on the development of minimally damaging electrode arrays, as well as on improved surgical techniques and the intra-operative application of steroids (for reviews see Pfingst et al. 2015; Nguyen et al. 2016; Dhanasingh and Jolly 2017; Khater and El-Anwar 2017; Bruce and Todt 2018). In contrast, there have been substantial advances that might have taken place anyway, using existing technology; examples include bilateral implantation, the provision of CIs to those with residual contralateral hearing, and the increased implantation of children and the reduction in the minimum age of implantation.

### (How) Can We Do Better?

#### Improving Hearing with Existing CI Technology and Methods

The end of the last century saw several major changes in the way CIs work: there was a switch from single-channel to multi-channel implants, from feature extraction to CIS and n-of-m strategies, and from quasi-analogue stimulation to the now-universal use of interleaved pulse trains (Eddington et al. 1978; Burian et al. 1979; Wilson et al. 1991; McDermott et al. 1992). Although no such step change has occurred in the last 20 years or so, it is clear from the studies described in the first four sections following the Introduction that some manipulations are likely to improve speech perception, even though the benefits are modest. Substantial improvements in medical devices can be achieved not only by revolution or breakthroughs, but also by the combination of incremental advances, and indeed this may be typical of mature technologies such as CIs. However, because each reported benefit has been at best modest, considerable care needs to be taken to determine what is really an advance and what is not. This is one reason why, when evaluating published studies, we have stressed the importance of basic aspects of experimental design such as blinding and the inclusion of adequate control groups or conditions; even small biases or experimenter effects may be of a similar size to the genuine advances one is trying to measure. Fortunately, attention to these basic tenets is now becoming the norm, for example with many recent studies incorporating a double-blind design (Magnusson 2011a; Koning and Wouters 2016; Nogueira et al. 2016; Riss et al. 2016; Bolner et al. 2020; Lopez-Poveda et al. 2020). Future improvements may build on the increased use in other areas of science and medicine of pre-registered reports (Munafò et al. 2017)—something that would be particularly useful in studies where there are multiple dependent variables and/or possible data analyses. This additional rigour will help researchers and companies alike to focus resources on those interventions that really work.

Another way of improving the realisation and evaluation of incremental benefits is to improve the power of each study by testing more participants or by confirming the findings using re-tests. Unfortunately, most studies of new techniques and fitting methods are limited to a dozen or so participants, partly due to limitations in researcher time and partly to the limited number of participants available in any one centre. In addition, researchers understandably focus on their own new method which they then evaluate using their own preferred tests, thereby complicating the comparison of the efficacy of different methods. A potential solution would be that, after obtaining preliminary evidence for the efficacy of their own method, different groups of researchers then perform a collaborative evaluation; this would provide a much-needed boost to participant numbers and allow the proposed innovations to be compared using the same group of participants and with the same testing materials and analyses. An intermediate solution would be to encourage more researchers to share their research software and test stimuli openly, in accordance with recent trends for open science, so that other researchers can include them as comparison conditions. Fortunately, the fact that CIs of a particular model are identical throughout the world makes this straightforward: one experimenter’s program should work in any laboratory without modification. Finally, it will be important to determine whether the advances obtained by different approaches are additive—an issue that led one scientist to remark, only half-jokingly, that they had seen so many 5% improvements reported throughout their career that patients should now be scoring more than 100% on speech tests. For example, the various processing algorithms that enhance modulations across time (Koning and Wouters 2016; Lamping et al. 2020) or across electrodes (Nogueira et al. 2016; Bolner et al. 2020; Lopez-Poveda et al. 2020) are likely addressing the same basic goal of increasing the contrast in the pattern of electrical stimulation (electrodogram), typically by attenuating or removing lower-amplitude pulses. To the extent that different algorithms attenuate the same pulses their benefits may not sum. Similarly, although the criteria used for different channel de-selection algorithms may reflect different aspects of neural health (Pfingst et al. 2015; Brochier et al. 2021), the need to retain some minimum number of channels of information for speech perception may make it impractical to combine them. In contrast, it might be possible to combine the advantages of temporal or spectral enhancement algorithms with either channel deactivation methods or with algorithms that enhance the representation of F0 in the electrodogram (Nogueira et al. 2016; Lopez-Poveda et al. 2017, 2020; Bolner et al. 2020). Furthermore, it may well be that pre-processing algorithms that increase the SNR at the input stage of the CI contribute somewhat independently to the improvements introduced by changes to the algorithm or by channel deactivation methods.

#### New Alternatives to Existing Technology

All of the research discussed so far has employed the now-traditional method of stimulating the AN using intra-scalar electrodes. Recently, there have been attempts to overcome the broad spread of excitation produced by this method by using alternative ways of stimulating the AN. One approach, originally adopted by Simmons et al. (1965) and recently refined and re-introduced by Middlebrooks and Snyder (2007), is to use intra-neural (IN) electrode arrays that directly penetrate the spiral ganglion. Using recordings from the cat IC, Middlebrooks and Snyder (2007) showed that, compared to MP stimulation of intra-cochlear electrodes, intra-neural (IN) stimulation required less current, produced sharper excitation patterns, and showed less interference between pairs of simultaneously stimulated electrodes. It has also been shown that IN stimulation can selectively access neurons that innervate the cochlear apex, thereby, it is argued, activating a brainstem pathway that is specialised for accurate temporal processing (Middlebrooks and Snyder 2010). An important future test for the feasibility of IN stimulation is that it should produce excitation patterns that are sharper than those for intra-scalar stimulation not only for MP mode, but also for more focused modes such as TP and AP. A comparison between excitation pattern widths for IN vs BP stimulation has indeed revealed some advantage for IN, although this depended somewhat on the exact method of comparing the excitation spread (Middlebrooks and Snyder 2007).

An alternative approach is to eschew electrical stimulation altogether by genetically manipulating spiral ganglion neurons, for example by local administration of adeno-associated viruses, so that they are responsive to light. Experiments using this optogenetic stimulation in rodents, recently reviewed by Dieter et al. (2020), have demonstrated successful optical activation of the auditory pathway by measuring the auditory brainstem response, multi-unit recordings from the auditory midbrain, and single-unit recordings from auditory cortex. An obvious advantage of optical over electrical stimulation is that it avoids current spread (as there is no current to spread) and, accordingly, leads to narrower neural excitation patterns. The temporal encoding of pulse rate with optical stimulation is not as good as with electrical or acoustic stimulation and depends strongly on the choice of light-sensitive protein, opsin, used (Keppeler et al. 2018; Dieter et al. 2020). One interesting approach is to combine electrical and optical stimulation, leading to (in the mouse inferior colliculus) improved spatial and temporal coding compared to optical stimulation alone (Thompson et al. 2020). Remaining challenges include the energy-efficient delivery of multi-channel optical stimulation to the AN, and the safety considerations surrounding gene therapy.

The efficacy of both optogenetic and penetrating-electrode stimulation is likely to be limited by the incomplete and sometimes patchy neural survival exhibited by human CI listeners, and optogenetic stimulation has so far been evaluated primarily in recently deafened animals. Uniform loss of spiral ganglion neurons may mean that there are not enough surviving neurons close to the stimulator to reach MCL, requiring stimulus levels to be increased so as to recruit more distant neurons. An optical or electrical stimulator located in a neural dead region may stimulate neurons both apical and basal to the stimulator, leading to a bimodal excitation pattern. These are the same issues that face focused modes of stimulation in conventional CIs (Litvak et al. 2007; Mesnildrey and Macherey 2015). We stress that this does not mean that there is no benefit in developing new tonotopically focused stimulation methods, and indeed penetrating array and optogenetic stimulation provide promising ways of doing so. However, it will be important to show that the substantial improvements in spatial selectivity that have been observed with these techniques in recently deafened animals lead to benefits under the conditions of neural degeneration that are typical of human CI listeners. In the far future, this issue may be alleviated by neurotrophin delivery designed to minimise or restore neural loss, as recently reviewed by Plontke et al. (2017) and by Pinyon et al. (2019).

### The Neural Basis for Success and Failure of Proposed Innovations

At present, innovations such as intra-neural and optogenetic stimulation have been tested only in animals. Similarly, recent developments that have been applied in humans, such as the use of focussed (e.g. TP and AP) stimulation and channel deactivation methods, have been motivated partly by physiological or histological experiments obtained from cats and guinea pigs (Bierer et al. 2010; George et al. 2014, 2015b). As noted in the “Focused and Current-Shared Stimulation” section, the effectiveness of these methods can vary across human participants or even between electrodes in the same participant, and some manipulations, such as focussed stimulation, appear to be more effective in animal experiments than for humans. Because one cannot obtain single-unit recordings from human participants, one usually has to rely on animal physiology to guide developments whose ultimate goal is to improve hearing by human CI listeners. This path is complicated by substantial differences in the stimuli used between the two types of study—for example, single-unit physiological experiments typically use single pulses whereas human experiments usually employ pulse trains, and the overall current level may differ between the two types of experiment. Further complications arise from the use of anaesthesia in the physiological studies (Chung et al. 2016), from the use of different outcome measures, such as behavioural judgements for humans and single-unit recordings for animals, and from between-species differences in the anatomy of the auditory nerve (Rattay et al. 2013). Recently, researchers have begun to bridge this gap by combining physiological experiments with threshold and supra-threshold behavioural measures using animals (Kadner and Scheich 2000; Vollmer et al. 2001; Pfingst et al. 2011; Benovitski et al. 2014; King et al. 2016; Rosskothen-Kuhl et al. 2021), and by developing electrophysiological measures of stimulus discrimination and cortical selectivity in humans that may in principle be applied to animal studies (Mathew et al. 2017, 2018; Presacco and Middlebrooks 2018; Carlyon et al. 2021).

Another promising avenue for understanding the neural and anatomical basis for human CI hearing comes from the increasingly sophisticated computational models of CI stimulation that have recently been developed (for a review see Kalkman et al. 2016). These in principle allow one to predict the success of novel stimulation methods and to understand why existing methods sometimes do and sometimes do not improve performance. To take one example, the model described by Kalkman (2015) predicts that the effectiveness of TP stimulation in producing narrower excitation patterns should depend both on the survival of the peripheral processes of the AN and on the electrode-modiolar distance. A publicly available version of each model would aid the development of new methods, the evaluation of existing ones, and the refinement of the models themselves; although some models are very complex, we believe that even simplified versions would prove useful when shared openly.

### Future Prospects

Prospects for improving hearing by CI listeners can, we think, be divided into three broad areas. One of these, which we have only mentioned briefly so far, concerns the small subset of patients who struggle to hear well (or sometimes at all) through their CIs. These include patients for whom CI stimulation elicits non-auditory sensations such as can be elicited by stimulation of the facial nerve, requiring some or all electrodes to be turned off or set to a maximum level that produces only a soft percept (Bahmer et al. 2010; Bahmer and Baumann 2016). Others obtain comfortably loud sensations on most or all electrodes but still struggle to hear well, and the reasons behind these instances of very poor performance remain incompletely understood (Firszt et al. 2004; Blamey et al. 2013; Boisvert et al. 2020). Our experience is that such patients rarely contribute to the cohort of participants who volunteer for often time-consuming experiments, and we suspect they are under-represented in at least some types of (e.g. psychophysical) studies, despite having the greatest potential (or at least room for) improvement.

For typically performing CI listeners, our improved understanding of the across- and within-listener variations in neural health and of the effects of electrode position, combined with the development of increasingly sophisticated computational models, raises the prospect of steadily improving modifications to the method of stimulation (such as variations in pulse shape and current-return path; Figs. 3 and 6). Rigorous evaluation of these methods and of novel, psychophysically grounded, signal processing algorithms—all of which may be applied in a patient-specific manner—may lead to sustained and gradual improvements in speech perception in the short to medium term. These improvements, combined with the gains already achieved this century, provide both a foundation for and a challenge to the identification of better methods of stimulating the auditory nerve and provide hope for more radical improvements in the long term. For example, as argued above, the effectiveness of both intra-neural and optogenetic stimulation is likely to depend on local neural survival and health, which, as described in the “Theoretical and Scientific Advances Resulting from CI Research” section, can now be measured using both behavioural and non-invasive electrophysiological techniques (Pfingst et al. 2015). The challenge arises because, as we succeed in improving hearing outcomes using conventional CI technology, the level of performance required of a new technology also increases. Whether the hare or the tortoise wins this race, we look forward to the further improvements in CI hearing obtained over the next 21 years.
